# Neoadjuvant versus definitive radiochemotherapy of locoregionally advanced oesophageal cancer—who benefits?

**DOI:** 10.1007/s00066-022-01929-y

**Published:** 2022-04-13

**Authors:** Christoph Schiffner, Hans Christiansen, Iris Brandes, Gerrit Grannas, Jörn Wichmann, Roland Merten

**Affiliations:** 1 Department of Radiation Oncology, Hannover Medical School, Carl-Neuberg-Str. 1, 30625 Hannover, Germany; 2 Institute for Epidemiology, Social Medicine and Health Systems Research, Hannover Medical School, Carl-Neuberg-Str. 1, 30625 Hannover, Germany; 3 Department of Visceral and Transplantation Surgery, Hannover Medical School, Carl-Neuberg-Str. 1, 30625 Hannover, Germany

**Keywords:** Oesophagectomy, Comorbidity, Subgroup analyses, Survival, Locoregional control

## Abstract

**Purpose:**

For years, there have been discussions on whether neoadjuvant radiochemotherapy followed by surgery (nRCT-S) is superior to definitive radiochemotherapy (dRCT) as the standard of care for locoregionally advanced oesophageal cancer (OC). This retrospective study aimed to evaluate our patient cohort regarding differences in survival and recurrence between nRCT‑S and dRCT.

**Methods:**

Data from 68 patients with dRCT and 33 patients with nRCT‑S treated from 2010 to 2018 were analysed. Comorbidities were recorded using the Charlson Comorbidity Index (CCI). Recurrence patterns were recorded as in-field or out-field. Kaplan–Meier analyses were used to compare survival data (overall survival [OS], progression-free survival [PFS], and locoregional control [LRC]).

**Results:**

Patients with nRCT‑S showed significantly lower CCI values than those with dRCT (*p* = 0.001). The median follow-up was 47 months. The median OS times were 31 months for nRCT‑S and 12 months for dRCT (*p* = 0.009), the median PFS times were 11 and 9 months, respectively (*p* = 0.057), and the median LRC times were not reached and 23 months, respectively (*p* = 0.037). The only further factor with a significant impact on OS was the CCI (*p* = 0.016). In subgroup analyses for comorbidities regarding differences in OS, the superiority of the nRCT‑S remained almost significant for CCI values 2–6 (*p* = 0.061).

**Conclusion:**

Our study showed significantly longer OS and LRC for patients with nRCT‑S than for those with dRCT. Due to different comorbidities in the groups, it can be deduced from the subgroup analysis that patients with few comorbidities seem to especially profit from nRCT‑S.

## Introduction

Oesophageal cancer (OC) is a moderately frequent tumour entity associated with a poor prognosis. In Germany, the incidence amounts to 9.7/100,000 for men and 2/100,000 for women, with 5‑year survival rates of approximately 18% for each [1]. The major histological subtypes are squamous cell carcinoma (SCC) and adenocarcinoma (AC), whereas the incidence of the latter is rising in the Western World and is associated with increasing rates of obesity [1].

In clinical practice, locoregionally advanced OC is regularly treated with neoadjuvant radiochemotherapy followed by surgery (nRCT-S) according to the CROSS protocol if patients are surgically and functionally operable [2–5]. However, the superiority of such a trimodal approach over definitive radiochemotherapy without surgery (dRCT) has been controversial for many years, including in randomized controlled trials [6–11]. Recently, two retrospective studies found no significant difference in overall survival [12, 13].

Nevertheless, there is still an urgent need for additional research data to improve evidence in the multimodal therapy of OC. Thus, the aim of our study was to evaluate our own patient collective regarding differences in survival and recurrence in the course of disease between nRCT‑S and dRCT.

## Patients and methods

**Fig. 1:**
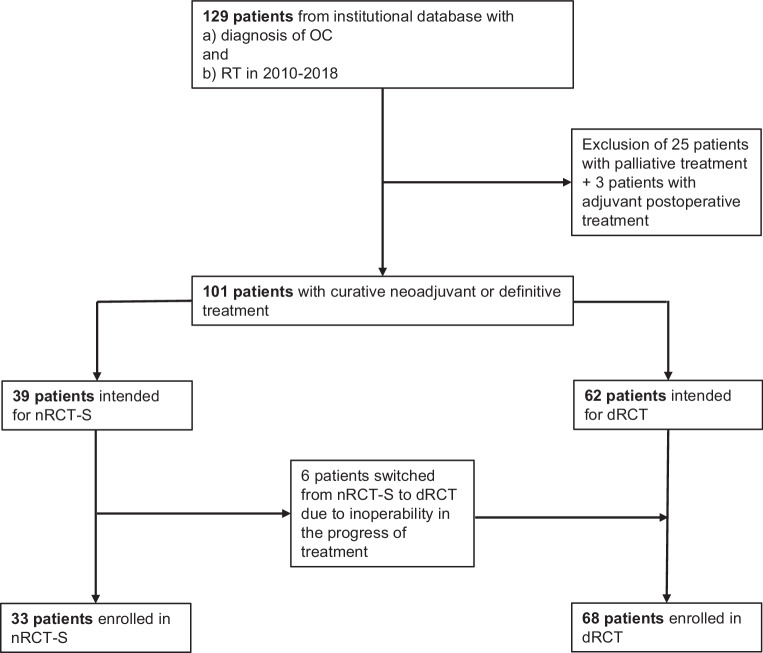
CONSORT diagram demonstrating patient selection. *OC* oesophageal carcinoma, *RT* radiotherapy, *nRCT‑S* neoadjuvant radiochemotherapy plus surgery, *dRCT* definitive radiochemotherapy

Our study retrospectively collected all patients with locoregionally advanced OC who underwent radiotherapy (RT) of the oesophagus between 2010 and 2018 at our radiation oncology department. The treatment concepts recorded included nRCT‑S and dRCT. Patients who received palliative or adjuvant postoperative treatment were excluded. Data were taken from our institutional databases and clinical information systems. In total, 101 patients were included, 33 receiving nRCT‑S and 68 receiving dRCT. Patient selection is demonstrated as a Consolidated Standards of Reporting Trials (CONSORT) diagram in Fig. 1.

After treatment, patients were regularly followed up every 3 months for 2 years and on a 6-monthly basis thereafter. Missing information about the disease course was obtained by contacting the patients’ oncologists, family doctors or other treating hospitals. The study was approved by the ethics committee of the Hannover Medical School (Nr. 9190_BO_K_2020), and all studies on patient data were carried out according to the principles of the Declaration of Helsinki. All patients provided consent for anonymous statistical analysis in advance. Every patient’s treatment decision was based on a multidisciplinary tumour board’s assessment. Determination of tumour localization and tumour staging included oesophagogastroscopy, endoscopic ultrasound (EUS), biopsy and computed tomography (CT) of the chest and abdomen. All patients were staged in accordance with the Union for International Cancer Control (UICC) classification system 8th edition [14]. The patients’ comorbidities were recorded using the Charlson Comorbidity Index (CCI) [15], whereby weights were assigned to the cancer itself. Nicotine use and alcohol consumption were evaluated as risk factors.

The chemotherapy (CTx) regimens administered were cisplatin + 5-fluorouracil (5-FU; cisplatin 20 mg/m^2^ + 5-FU 1000 mg/m^2^ over 5 days at weeks 1 and 5) as per the Herskovic protocol [16] and later paclitaxel + carboplatin (paclitaxel 50 mg/m^2^ + carboplatin 2 mg × ml^−1^ × min^–1^ area under the curve up to a total of five cycles at weekly intervals). The RT technique used was three-dimensional conformal radiotherapy (3D-CRT), contouring the target volume and organs at risk. Treatment planning of all patients was performed in compliance with the Quantitative Analyses of Normal Tissue Effects in the Clinic (QUANTEC) dose constraints for organs at risk (e.g., V_20Gy_ < 30% for both lungs and V_30Gy_ < 46% for the heart) [17]. The tumour and affected locoregional lymph nodes visible on CT were decisive for the gross tumour volume (GTV). Adding margins (radial: 1 cm, longitudinal: 3 cm) to the GTV created the clinical target volume (CTV), followed by another margin of 0.8 cm creating the planning target volume (PTV) to compensate for setup errors. Dose delivery was performed according to the International Commission on Radiation Units and Measurements (ICRU) reports [18, 19]. Patients were examined by oesophagoscopy 4 weeks after completion of dRCT. In the case of macroscopic persistence of the tumour, an additional boost of 8 Gy in two fractions of high-dose-rate brachytherapy was applied. Brachytherapy was performed after CT-based three-dimensional planning to cover 95% of the GTV of the persistent tumour with 95% of the prescribed dose. 192-Iridium was used in an afterloading technique. For brachytherapy, the Bonvoisin–Gérard oesophageal applicator set and the microSelectron® Digital afterloading platform were used (both Elekta, Stockholm, Sweden).

Since 2012, patients who received nRCT‑S have been treated as per the CROSS protocol with 41.4 Gy and paclitaxel + carboplatin [3]. Previously, those patients mainly received doses of 46 Gy and cisplatin + 5-FU. Regarding the histopathological response to radiochemotherapy (RCT) on the basis of the intraoperatively taken tissue samples, patients who received nRCT‑S were classified into “complete tumour regression” and “incomplete tumour regression” [20]. Patterns of failure in the course of disease were recorded as either in-field or out-field (the latter included distant metastases).

### Statistics

Descriptive analyses were performed by calculating the median or mean for continuous variables and by determining counts and percentages for categorical data. Regarding patient characteristics, the two treatment groups were compared using Student’s *t*-test for continuous variables, χ^2^ test for yes/no-specifications and the Mann–Whitney U test for other ordinal and categorical data.

Kaplan–Meier and log-rank analyses were used to estimate and compare survival data such as overall survival (OS), progression-free survival (PFS), locoregional control (LRC) and time to distant failure (TDF). PFS was calculated, including death without progression as an event, similar to progression itself, according to the current U.S. Food and Drug Administration (FDA) guidance [21]. For LRC and TDF, death without local and distant progression was censored according to Machtay et al. [22]. The method of Schemper and Smith was used to measure follow-up times [23]. *P* values < 0.05 were considered significant, and the statistical analyses were performed using IBM SPSS v27 software (IBM Corp., Armonk, NY, USA).

## Results

### Patient and tumour characteristics

An overview of patient characteristics is given in Table 1. Both treatment groups were well balanced in terms of age, sex, histology and grading. In total, the mean age was 64.3 years, 78.2% were men, 70.3% had SCC (26.7% AC, 3.0% small cell carcinoma) and 65.3% had G2 disease (33.7% grading G3, 1% grading G1).

**Table 1:** Patient Characteristics

	All patients	nRCT‑S (*n* = 33)	dRCT (*n* = 68)	*p*-value
*Therapy intended*	101	39	62	–
*Therapy performed*	101	33	68	–
*Age, years*				
Mean	64.3	62.3	65.2	0.220
Median	65	63	67	
Range	34–89	34–80	37–89	
*Sex*				
Male	79 (78%)	27 (82%)	52 (77%)	0.543
Female	22 (22%)	6 (18%)	16 (24%)	
*Charlson Comorbidity Index (CCI)*				
Mean	5.7	4.6	6.1	0.001*
Median	6	4	6	
Range	2–14	2–8	3–14	
*Clinical tumour staging*				
cT1	3 (3%)	0 (0%)	3 (5%)	0.191
cT2	18 (18%)	2 (6%)	16 (24%)	
cT3	65 (65%)	28 (85%)	37 (55%)	
cT4	14 (14%)	3 (9%)	11 (16%)	
Not available, *n*	1	0	1	
*Clinical nodal staging*				
cN0	28 (28%)	8 (24%)	20 (29%)	0.586
cN+	73 (72%)	25 (76%)	48 (71%)	
– cN1	53 (53%)	18 (55%)	35 (52%)	
– cN2	17 (17%)	7 (21%)	10 (15%)	
– cN3	3 (3%)	0 (0%)	3 (4%)	
*Clinical metastasis staging*				
cM0	92 (91%)	32 (97%)	60 (88%)	0.148
cM1	9 (9%)	1 (3%)	8 (12%)	
*UICC stage*				
I	2 (2%)	0 (0%)	2 (3%)	0.806
II	18 (19%)	4 (13%)	14 (22%)	
III	56 (58%)	23 (72%)	33 (51%)	
IV	21 (22%)	5 (16%)	16 (25%)	
Not available, *n*	4	1	3	
*Histology*				
Squamous cell carcinoma	71 (70%)	22 (67%)	49 (72%)	0.591
Adenocarcinoma	27 (27%)	10 (30%)	17 (25%)	
Small cell carcinoma	3 (3%)	1 (3%)	2 (3%)	
*Grading*				
Well differentiated (G1)	1 (1%)	0 (0%)	1 (2%)	0.962
Moderately differentiated (G2)	66 (65%)	22 (67%)	44 (65%)	
Poorly differentiated (G3)	34 (34%)	11 (33%)	23 (34%)	
*Location in oesophagus*				
Upper third	7 (7%)	0 (0%)	7 (10%)	0.031*
Middle third	18 (18%)	4 (12%)	14 (21%)	
Lower third	76 (75%)	29 (88%)	47 (69%)	
*Chemotherapy*				
Yes	86 (85%)	33 (100%)	53 (78%)	0.003*
No	15 (15%)	0 (0%)	15 (22%)	
*Chemotherapy completed*				
Yes	49 (57%)	19 (58%)	30 (57%)	0.929
No	37 (43%)	14 (42%)	23 (43%)	
*Chemotherapy regimen*				
Carboplatin + paclitaxel	48 (56%)	17 (52%)	31 (59%)	0.588
Cisplatin + 5-FU	30 (35%)	13 (39%)	17 (32%)	
Others	8 (9%)	3 (9%)	5 (9%)	
*Days from end of RT to operation*				
Mean	42.7	42.7	–	–
Median	41	41	–	–
Range	13–103	13–103	–	–

Values are expressed as median (range) or *n* (%)

*nRCT‑S* neoadjuvant radiochemotherapy plus surgery, *dRCT* definite radiochemotherapy, *UICC* Union for International Cancer Control, *RT *radiotherapy

*Statistically significant *p*-value

Staging showed no significant differences (for UICC staging *p* = 0.806). However, patients with cT3-stage disease were proportionately more frequently assigned to nRCT‑S (84.8% within nRCT‑S vs. 55.2% in dRCT; for whole cT staging *p* = 0.191). These patients showed lower mean CCI values (5.3) than those with cT2-stage and cT4-stage disease (6.2 and 6.2, respectively). Patients with stage cM1 disease received dRCT (11.8%, *n* = 8, within the dRCT vs. 3.0%, *n* = 1, in the nRCT‑S group; *p* = 0.148). These patients were oligometastasized and were therefore treated with curative intent. The oligometastases were treated by RT (*n* = 7) or by surgery and RT (*n* = 2).

Patients with higher-grade comorbidities were significantly more likely to be assigned to dRCT (mean CCI in dRCT = 6.1 vs. nRCT-S = 4.6, *p* = 0.001). Proximal oesophageal tumours were all treated with dRCT, whereas tumours in the lower third were proportionately treated with nRCT‑S (lower third in dRCT 69.1% vs. nRCT‑S 87.9%, *p* = 0.031).

### Treatment details

Thirty-three patients (32.7%) received nRCT‑S (initially intended: *n* = 39), and 68 patients (67.3%) were treated by dRCT (initially intended: *n* = 62). The RCT differed in terms of the application of CTx (applied in dRCT 77.9% vs. nRCT‑S 100.0%, *p* = 0.003) and the mean cumulative RT dose (dRCT 56.4 Gy vs. *n*-RCT‑S 42.8 Gy, *p* < 0.001). The CTx regimes were distributed approximately equally as was the completeness of CTx (58% of patients completed full-dose CTx in the nRCT‑S group and 57% completed full-dose CTx in the dRCT group). All others had to undergo reductions in dosage due to toxicity or comorbidities. Brachytherapy was only applied with dRCT (11.8%). In nRCT‑S, the mean time between the end of RT and the operation was 42.7 days. In the histopathological analysis, out of 33 patients receiving nRCT‑S, 9 (27.3%) showed complete tumour regression and 24 (72.7%) showed partial regression.

### Survival

**Fig. 2:**
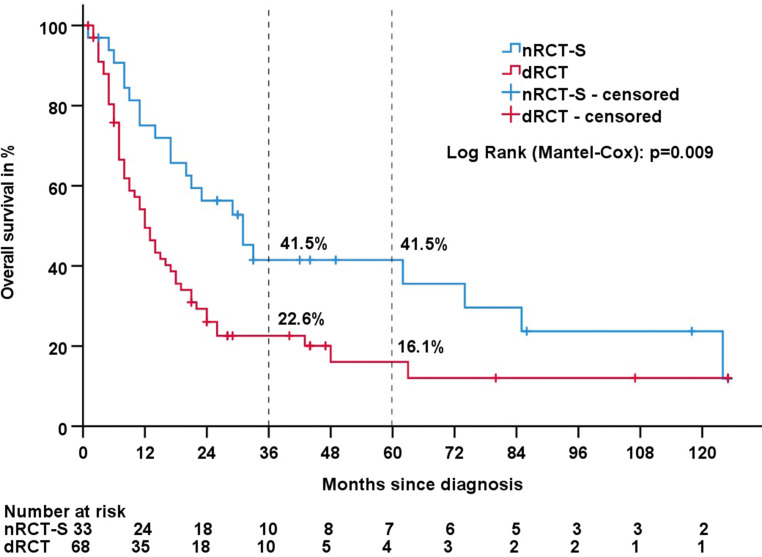
Kaplan–Meier estimates of overall survival for nRCT‑S vs. dRCT. *nRCT‑S* neoadjuvant radiochemotherapy plus surgery, *dRCT* definitive radiochemotherapy

Details on survival data are shown in Tables 2 and 3. The median follow-up for all patients was 47 months. The median OS times were 31 months in the nRCT‑S group and 12 months in the dRCT group (*p* = 0.009; Kaplan–Meier analysis shown in Fig. 2). The 3‑year survival rates were 28.6% for all patients, 41.5% for nRCT‑S and 22.6% for dRCT. The only other factor with a significant influence on OS was the CCI (*p* = 0.016; for the compared subgroups in Table 2: *p* = 0.012). Factors such as age, staging and histology had no significant impact on OS. The median OS times after nRCT‑S were 74 months in patients with complete tumour regression and 29 months in patients with partial regression (*p* = 0.289).

**Table 2:** Data for overall survival (OS) and locoregional control (LRC)

	OS, months; median (range)	3‑year OS, %	*p*-value (for OS)	LRC, months; median (range)	*p*-value (for LRC)
*All patients*	17 (1–125)	28.6	–	73 (1–125)	–
*Treatment groups*					
nRCT‑S	31 (1–125)	41.5	0.009*	n.r. (6–125)	0.037*
dRCT	12 (2–125)	22.6		23 (1–125)	
*Tumor regression grade (within the nRCT‑S group)*					
Complete regression	74 (8–118)	55.6	0.289	n.r. (73–118)	0.096
Partial regression	29 (1–125)	35.3		61 (6–125)	
*Clinical tumour staging*					
cT1-cT2	26 (2–86)	40.7	0.314	n.r. (3–86)	0.151
cT3	17 (1–125)	27.4		61 (2–125)	
cT4	9 (3–124)	21.4		15 (1–124)	
*Clinical nodal staging*					
cN0	18 (4–125)	29.7	0.511	73 (2–125)	0.479
cN1-cN3	15 (1–125)	28.4		61 (1–125)	
*Clinical metastasis staging*					
cM0	17 (1–125)	30.8	0.112	73 (1–125)	0.484
cM1	9 (5–33)	0.0		11 (4–33)	
*UICC stage*					
I	No death recorded	–	0.326	5 (5–44)	0.752
II	17 (7–125)	29.4		73 (5–125)	
III	17 (1–125)	28.1		61 (2–125)	
IV	11 (3–124)	22.9		15 (1–124)	
*Histology*					
Squamous cell carcinoma	12 (1–125)	32.6	0.758	73 (1–125)	0.921
Adenocarcinoma	20 (2–43)	16.6		21 (2–43)	
*Grading*					
G1–G2	17 (1–125)	31.1	0.457	61 (2–125)	0.512
G3	16 (3–107)	23.5		n.r. (1–107)	
*Charlson Comorbidity Index (CCI)*					
2–4	31 (1–125)	44.2	0.012*	73 (2–125)	0.096
5–6	13 (2–107)	22.8		n.r. (1–107)	
7–14	12 (2–33)	17.5		17 (3–33)	
*Age, years*					
34–55	21 (4–118)	37.7	0.433	n.r. (3–118)	0.522
56–65	14 (1–125)	38.1		61 (1–125)	
66–75	17 (3–125)	24.2		21 (3–125)	
75–89	17 (2–42)	11.2		n.r. (6–29)	

*nRCT‑S* neoadjuvant radiochemotherapy plus surgery, *dRCT* definite radiochemotherapy, *UICC* Union for International Cancer Control, *n.r.* median not reached

*Statistically significant *p*-value

**Table 3:** Survival data for nRCT‑S vs dRCT

	All patients (*n* = 101)	nRCT‑S (*n* = 33)	dRCT (*n* = 68)	*p*-value
*Follow-up, months*	47 (1–125)	86 (3–125)	44 (1–125)	0.245
*Overall survival, months*	17 (1–125)	31 (1–125)	12 (2–125)	0.009*
*Disease-specific survival, months*	26 (3–125)	62 (8–125)	18 (3–125)	0.020*
*Progress in the course of disease*				
Yes	59 (58%)	18 (55%)	41 (60%)	0.582
No	42 (42%)	15 (46%)	27 (40%)	
*Progression-free survival, months*	10 (1–125)	11 (1–125)	9 (1–125)	0.057
*Local recurrence in the course of disease*				
Yes	37 (37%)	9 (27%)	28 (41%)	0.174
No	64 (63%)	24 (73%)	40 (59%)	
*Locoregional control, months*	73 (1–125)	n.r. (6–125)	23 (1–125)	0.037*
*Distant metastasis in the course of disease*				
Yes	38 (38%)	15 (46%)	23 (34%)	0.258
No	63 (62%)	18 (55%)	45 (66%)	
*Freedom of distant metastasis, months*	36 (2–125)	36 (5–125)	29 (2–125)	0.876
*Death within 30 days after completion of RT*				
Yes	2 (2%)	1 (3%)	1 (2%)	0.598
No	99 (98%)	32 (97%)	67 (99%)	
*Death within 90 days after completion of RT*				
Yes	9 (9%)	1 (3%)	8 (12%)	0.148
No	92 (92%)	32 (97%)	60 (88%)	

Values are expressed as median (range) or *n* (%)

*nRCT‑S* neoadjuvant radiochemotherapy plus surgery, *dRCT* definite radiochemotherapy,* n.r.* median not reached, *RT* radiotherapy

*Statistically significant *p*-value

**Fig. 3:**
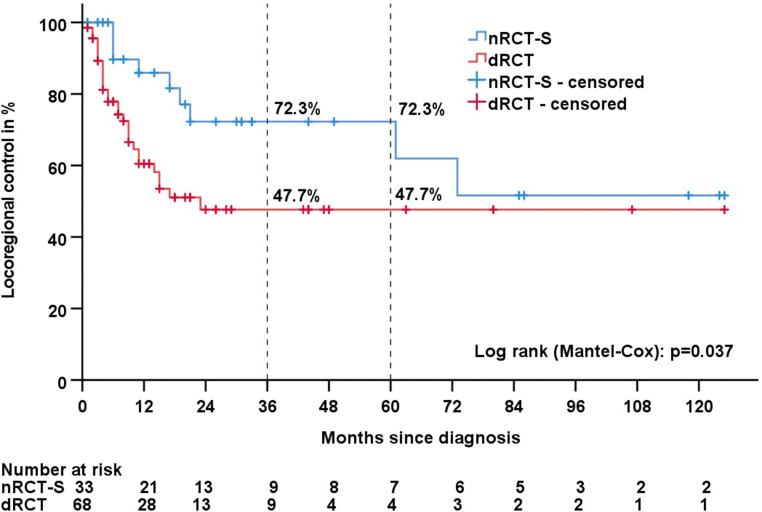
Kaplan–Meier estimates of locoregional control for nRCT‑S vs. dRCT. *nRCT‑S* neoadjuvant radiochemotherapy plus surgery, *dRCT* definitive radiochemotherapy

The median disease-specific survival times were 62 months in the nRCT‑S group and 18 months in the dRCT group (*p* = 0.020). Tumour progression occurred in 18 (54.5%) patients in the nRCT‑S and 41 (60.3%) patients in the dRCT group. The median PFS times were 11 months in the nRCT‑S group and 9 months in the dRCT group (*p* = 0.057). Local recurrence occurred in 9 (27.3%) nRCT‑S patients compared with 28 (41.2%) dRCT patients. The median LRCs were not reached for nRCT‑S and 23 months for dRCT (*p* = 0.037; Kaplan–Meier analysis shown in Fig. 3). Distant metastasis occurred in 15 (45.5%) patients in the nRCT‑S group and 23 (33.8%) patients in the dRCT group. The median TDF times were 36 months for nRCT‑S and 29 months for dRCT (*p* = 0.876).

Deaths within 30 days (D30) after treatment completion occurred in 1 (3.0%) patient in the nRCT‑S group (perioperative mortality = 3.0%) and 1 patient (1.5%) in the dRCT group (*p* = 0.598). Deaths within 90 days (D90) occurred in 1 (3.0%) patient in the nRCT‑S group and 8 patients (11.8%) in the dRCT group (*p* = 0.148).

### Subgroup analyses

In analyses within subgroups of CCI regarding differences in OS between nRCT‑S vs. dRCT, the superiority of nRCT‑S tended to be significant in CCI values 2–6 (*p* = 0.061). This subgroup contained 31 of 33 (93.9%) patients treated with nRCT‑S and 45 of 68 (66.2%) patients receiving dRCT. Within this subgroup, the CCI did not differ significantly between the groups (mean CCI in nRCT-S = 4.5 vs. dRCT = 4.9, *p* = 0.174).

### Toxicity

Events of acute toxicity of grades 3 and 4 during RCT are shown in Table 4. No events of grade 5 were recorded. Acute oesophagitis tended to occur in more patients in the dRCT approach than in the nRCT‑S approach (*p* = 0.084). Late toxicities were grade 0 in 50.6% of patients recorded. Grade 1–2 toxicities occurred in 45.5% of patients, grade 3 in 3.9% and grade 4 toxicity did not occur.

**Table 4:** Toxicity events of grade 3 and 4 according to CTCAE

	All patients (*n* = 101)	nRCT‑S (*n* = 33)	dRCT (*n* = 68)	*p*-value
Esophagitis	15 (15%)	2 (6%)	13 (19%)	0.084
Leukopenia	25 (25%)	8 (24%)	17 (25%)	0.934
Pneumonitis	1 (1%)	0 (0%)	1 (2%)	0.484

*nRCT‑S* neoadjuvant radiochemotherapy plus surgery,* dRCT* definite radiochemotherapy,* CTCAE* Common Terminology Criteria for Adverse Events

## Discussion

Our study showed significantly longer OS and LRC for patients with oesophageal cancer receiving surgery after neoadjuvant radiochemotherapy than for those receiving definitive radiochemotherapy without surgery. However, the CCI values in the latter group were significantly higher. Nevertheless, in a subgroup of lower CCI values (2–6), the superiority of nRCT‑S regarding OS still trended to be significant. Regarding the differently distributed patient characteristics, few patients with cT2- and cT4-stage disease were treated with nRCT‑S due to the relatively high-grade comorbidities in our patients with cT2-stage disease—as low-risk lesions would otherwise rather have been treated by surgery only without any neoadjuvant therapy [24]—and due to the inoperability of the tumour itself in most of our patients with cT4-stage disease. Proximal cervical tumours were all treated with dRCT because of known operational difficulties [25]. Our study showed no significant differences in OS between the histological subtypes of SCC and AC, probably caused by the small number of cases. In the literature, a longer OS for SCC is regularly described [3].

To show the whole patient population treated with curative intent, we did not set inclusion criteria such as performance status limits or freedom of any metastasis (as we included curatively treated oligometastases), as other retrospective analyses did [12]. This might have led to lower survival rates for dRCT in our study compared to other data, while our nRCT‑S rates are similar or even higher [10–13]. However, survival rates range widely in the literature, probably depending on the study design. In prospective studies, Stahl et al. and Bedenne et al. [10, 11] showed equal results for both treatment approaches, with median OS rates that were considerably lower for nRCT‑S than in our study (16.4 and 17.7 months in nRCT‑S, 14.9 and 19.3 months in dRCT, respectively). In contrast, with a median OS of 49.4 months for patients receiving nRCT‑S, remarkably high survival rates were shown in the CROSS trial [3], making the applied dose of 41.4 Gy the current standard for nRCT‑S. A similarly high median OS of 45 months for nRCT‑S has been shown in a retrospective study by Vitz et al. [26].

Likewise, for median PFS, wide ranges can be found, from 11 to 15.6 to 37.7 months (each for nRCT‑S, in our study, in Haefner et al. [12] and in the CROSS trial [3], respectively). Regarding toxicity, the rate of occurrence of acute adverse events of grades 3 and 4 is mostly described as 10–20% of patients [3, 11, 12], similar to our results. Among our patients, events of acute toxicity occurred more frequently in dRCT, probably due to the higher radiation doses applied.

In the nRCT‑S approach, no considerable incidence of D30 was seen. This is consistent with the finding of Dähn et al. that RT does not cause an increase in perioperative mortality if modern RT techniques are used and certain constraints are consistently followed in RT planning [27].

In terms of CTx, the dRCT group included patients not receiving any CTx because of high-grade comorbidities, underscoring the differences in general health constitution between the treatment groups. Similarly, compliance with full-dose CTx was lower in our study than in other studies [10, 12, 13] due to the high incidence of comorbidities.

There is an ongoing debate about the ideal treatment for locoregionally advanced OC in the literature. Regarding the RT dose for nRCT‑S, Paireder et al. recently considered a modification of the predominant CROSS regimen up to a dose of 46 Gy without disadvantages in safety and effectiveness [3, 28]. For dRCT, de Vos-Geelen et al. emphasized maintaining the dose to ≤50.4 Gy to ensure less toxicity and comparable effectiveness [29], which was confirmed by the randomized controlled ARTDECO study published in 2021 [30]. Moreover, the continuation of treatment for patients with complete remission (CR) after neoadjuvant RCT remains uncertain. While some publications consider CR to be an indication for omitting the operation and instead performing a dose escalation of RCT, others still prefer surgery [6, 31]. This possibly multistep approach raises the question of how response can be measured in routine clinical practice. The current multicentre randomized SANO study compares surgery versus active surveillance for patients with CR [32, 33]. The diagnostic methods used in this study include endoscopy with biopsies in the first evaluation after nRCT‑S and 18F-fluorodeoxyglucose positron emission tomography/computed tomography (18F-FDG-PET-CT) followed by endoscopy with biopsies in the second evaluation [32]. A retrospective study in 2018 proposed 18F-FDG-PET-CT as a sufficient method for response evaluation in routine clinical practice, as it combines metabolic and morphologic evaluations [34]. In contrast, a meta-analysis pointed out that noninvasive imaging, such as CT, PET-CT and EUS, is not sufficiently precise to identify CR [35]. In our department, thus far, the histopathological examination of the intraoperatively taken tissue samples was the only evaluation of response. Within this study, a significantly longer median OS in patients with complete tumour regression was not reached due to the small number of patients in this subgroup. However, research is ongoing in this field, and the results of current prospective studies, such as the SANO study or the PRIDE study, could yield additional findings [32, 36]. Another discussed therapeutic option is salvage surgery, which is considered a valuable approach for patients with persistent or recurrent disease after dRCT or in a stage of active surveillance, as mentioned above. In a large multicentre study published in 2015, salvage surgery after dRCT led to similar OS when compared to nRCT‑S [37].

In summary, it is of utmost importance to choose patients carefully for either nRCT‑S or dRCT. The assessment should include at least age and comorbidities, tumour characteristics and, ideally, clinical response to RCT. For elderly patients it is advisable to consult a geriatric physician within the multidisciplinary tumour board [38]. The contribution of our study to the discussion regarding the optimal treatment for locoregionally advanced OC emphasizes that patients with none or few comorbidities seem to profit from nRCT‑S. However, the trend in recent literature has been towards equal effectiveness of dRCT [10–13].

The retrospective design and the size (*n* = 101) of the study caused limitations in the statistical analysis. Hence, conclusions should be drawn with caution. Nevertheless, the results are worth considering, as such data are rare in this field and, in contrast to randomized prospective studies, retrospective analyses give an authentic representation of the patient population in daily clinical routine. Finally, further research from different perspectives of multimodal therapy is needed to improve the outcomes of patients with oesophageal cancer.

## Conclusion

Our study showed significantly longer overall survival and locoregional control for patients treated with neoadjuvant RCT followed by surgery than for those receiving definitive RCT, especially for subgroups of patients with few comorbidities; however, all of these studies were subject to the aforementioned limitations. In contrast, recent literature has trended towards the equal effectiveness of both approaches. Further research is needed to clarify the standard of care as well as to establish technical developments and multistep approaches, including active surveillance.
